# Evaluating the effect of pupil dilation on spectral-domain optical coherence tomography measurements and their quality score

**DOI:** 10.1186/s12886-015-0168-y

**Published:** 2015-12-11

**Authors:** Lucia Tanga, Gloria Roberti, Francesco Oddone, Luciano Quaranta, Manuela Ferrazza, Francesca Berardo, Gianluca Manni, Marco Centofanti

**Affiliations:** IRCCS-Fondazione GB Bietti, Via Livenza 3, 00198 Rome, Italy; DSMC, Università degli studi di Brescia USVD “Centro per lo studio del Glaucoma”, P.le Spedali Civili, 1 - 25123, Brescia, Italy; DSCMT, Università di Roma Tor Vergata, Viale Oxford 81, 00133 Rome, Italy

## Abstract

**Background:**

Spectral-domain optical coherence tomography (SD-OCT) provides fast scan speed and high scan resolution improving its diagnostic accuracy. The purpose of this study was to evaluate if SD-OCT measurements and their quality score are influenced by pupil dilation.

**Methods:**

Retinal nerve fiber layer thickness (RNFL), ganglion cell complex (GCC) and optic nerve head (ONH) were measured in one eye of 57 glaucoma patients and 36 healthy subjects using spectral domain optical coherence tomography (SD-OCT) before and after pupil dilation. Comparisons were made between measurements and their quality score pre- and post dilation (Signal Strength Index, SSI). Overall RNFL, average GCC and ONH rim volume were considered in the analysis.

**Results:**

No statistically significant differences were found between pre- and post-dilation measurements in both groups (glaucoma: RNFL 80 ± 15 μm vs 80 ± 16 μm, *p* = 0.87; GCC 81.35 ± 13.4 μm vs 81.10 ± 13.14 μm, *p* = 0.92; ONH 0.05 ± 0.11 mm^3^ vs 0.04 ± 0.07 mm^3^, *p* = 0.74; controls RNFL 99 ± 12 μm vs 98 ± 14 μm, *p* = 0.70; GCC 92.12 ± 6.7 μm vs 91.54 ± 7.05 μm, *p* = 0.72; ONH 0.11 ± 0.1 mm^3^ vs 0.04 ± 0.07 mm^3^, *p* = 0.36) nor between pre- and post-dilation quality score (glaucoma SSI RNFL 54.3 ± 10.3 vs 51.7 ± 18.1, *p* = 0.12; SSI GCC 58 ± 9.5 vs 57 ± 8.09, *p* = 0.55; SSI ONH 48.5 ± 7.6 vs 46.6 ± 7.2, *p* = 0.16; controls SSI RNFL 57 ± 10.3 vs 54 ± 9.31, *p* = 0.2; SSI GCC 60.9 ± 8.1 vs 58.8 ± 7.3, *p* = 0.3; SSI ONH 51.5 ± 8.9 vs 50.4 ± 8.3, *p* = 0.59).

**Conclusion:**

Pupil dilation doesn’t affect SD-OCT measurements and their quality score.

## Background

Optical coherence tomography (OCT) is a non-invasive method of imaging the optic nerve head (ONH) and retina and it has been shown to provide reproducible measures of both ONH morphometry and retinal nerve fibre layer (RNFL) thickness, playing a growing role in the diagnosis and monitoring of glaucoma [1–8].

The spectral-domain OCT technology (SD-OCT), has replaced the time-domain OCT (TD-OCT) providing faster scan speed, improving scan resolution and potentially increased diagnostic accuracy [9, 10]. The RTVue-100 SD-OCT (Optovue Inc., Fremont, California, USA) is a commercially available SD-OCT with an axial resolution of 5 μm in tissue and a scan speed of 26000 A-scans/s. RTVue OCT is also equipped with a proprietary segmentation algorithm aimed at measuring the ganglionar cell complex (GCC) thickness within the macular region [11]. When introducing a device in the clinical practice, it is important to assess its reproducibility, its diagnostic accuracy and the factors that might influence these two features, like media opacities and pupil dilation. Although the manufacturer of the RTVue-100 indicates the quality score limit to accept the images (the Signal Strenght Index, SSI), there is no study in literature investigating the effects of pupil dilation on it. Low signal strength can result in poor image resolution, lack of retinal detail, and an increase in segmentation errors (because there is little image structure for the algorithms to use in segmenting the various layers). Thus, the purpose of this study was to evaluate the influence of pupil dilation on RTVue-100 RNFL, ONH and GCC measurements and their quality in glaucoma patients and healthy subjects.

## Methods

The study was conducted at the IRCCS-Fondazione GB Bietti, Rome (Italy) according to the principles of the Declaration of Helsinki and the ethics committee of the institution that approved the protocol. Written informed consent was obtained from all enrolled subjects.

Two groups of people were involved, glaucoma patients and healthy subjects as control group.

Glaucoma was defined as the presence of a repeatable visual field (VF) defect, commensurate with optic nerve damage. A glaucomatous VF change was defined as the consistent presence of a cluster of three or more non-edge points on the pattern deviation plot with a probability of occurring in <5 % of the normal population with one of these points having the probability of occurring in <1 % of the normal population, a pattern standard deviation with *p* < 5 %, or a glaucoma hemifield test result outside normal limits. VF defects had to be reliable (false positive <15 %; fixation losses and false-negative responses <25 %) and confirmed in at least two tests no more recent than one month.

Healthy subjects had to have intraocular pressure of less than 22 mmHg, normal-appearing optic disc and normal VF test result.

Participants in both groups were excluded if they had spherical refractive error greater than ±6 diopters, astigmatism greater ±3 diopters, any active or past retinal pathologies (including diabetic retinopathy or age-related macular degeneration), opacities of optic media that could bias functional and structural testing, history of ocular surgery (except for uncomplicated cataract or glaucoma surgery), use of miotic drugs as well as pupils smaller than 3 mm.

People were selected among patients and their relatives or partners referring at glaucoma department of IRCCS-Fondazione GB Bietti and matched by age.

All patients underwent a complete ophthalmological examination including visual acuity measurement, slit lamp examination, tonometry with the Goldmann applanation tonometer, gonioscopy, indirect ophthalmoscopy assessment of the optic nerve head with a 90D lens and automated perimetry (24–2 SITA Standard on Humphrey Visual Field Analyzer; Carl Zeiss Meditec Inc., Dublin, CA, USA) to determine eligibility.

Pupil size was then automatically measured by OPD-Scan II Nidek Optical Path Difference Scanning System (Fremont, California) before and 30 min after pupil dilation obtained by one drop of tropicamide 1 %. It is important to highlight that RTVue-100 doesn’t give enough light to cause significant constriction during scan acquisition. Infact, it uses a scanning laser diode to emit a scan beam with a wavelength of 840 ± 10 nm to provide images of ocular microstructures.

All subjects underwent RNFL, ONH and GCC assessment with the RTVue-100 OCT before and after pupil dilation. Subjects were seated with the chin comfortably positioned on a chin rest and the machine properly aligned. The subject was then instructed to look at the internal fixation target to bring the optic nerve head or the macula (for GCC scan protocol) within view of the examiner. The position of the aiming circle was adjusted by the operator to match the optic nerve head best focus and centralization. For all scan sections we have performed an automatic Axial length (“Auto Z”), Focus (“Auto F”) and to offset the refractive condition, and “Polarization (“Auto P”) as recommended by the manufacturer [11]. The same experienced operator performed all the measurements before and after pupil dilation.

RNFL overall, GCC average and ONH rim volume measurements were collected for this study.

Image quality on the RTVue-100 is determined by SSI parameter which is a measure of the average signal strength across the scan, so that the stronger the OCT signal, the higher the SSI value.

The SSI range from near 0 (no signal) to approximately 90 (very strong signal). The general guidelines from the manufacturer are as follows: SSI of less than 30 is a very poor quality scan that cannot be analyzed, SSI between 30 and 40 is a poor quality scan that can be analyzed but should be retaken to improve if possible, SSI of more than 40 but less than 50 is an adequate quality scan that can be analyzed, SSI of more than 50 but less than 60 is a good quality scan, and SSI of more than 60 is a very good quality scan [11].

All images acquired were included and SSI RNFL, SSI GCC and SSI ONH values were collected for this study.

### Statistical analysis

Continuous data were expressed by mean and standard deviation (SD) and paired *t*-test or Wilcoxon sign rank test were used to evaluate differences between measurements and between SSI pre- and post- dilation.

To investigate the possible influence of the quality score on the variability between pre- and post-dilation measurements, we evaluated the correlation between this variability and the mean quality score of the two measurements, using Pearson’s correlation coefficient.

The analysis was performed using JMP 7.0 (SAS Institute Inc. Cary, NC, USA).

## Results

Fifty-seven eyes of 57 glaucoma patients and 36 eyes of 36 age matched healthy subjects were included in the study (55 ± 14.2 years versus 57 ± 14.9 years, *p* = 0.63).

As expected, patients had worse VF defect compared to healthy subjects (MD −7.35 ± 8 dB versus −0.9 ± 1.2 dB, *p* < 0,001 and PSD 5.11 ± 3.52 dB versus 1.56 ± 0.36 dB, *p* < 0.001), while refraction didn’t differ between the two groups (−1.43 ± 3.5 diopters versus −1.37 ± 3.18 diopters, *p* = 0.94). BCVA was very good in both groups (0.0 logMAR).

Patients had average pupil size of 4.38 ± 0.92 mm before pupil dilation and 6.96 ± 0.80 mm after pharmacologically induced mydriasis, while healthy subjects had average pupil size of 4.50 ± 0.70 mm pre-dilation and 7.01 ± 0.80 mm post-dilation (*P* = 0.95).

RNFL overall, GCC average and ONH rim volume measurements in both groups didn’t differ statistically, as presented in Table 1.

**Table 1:** Student *t*-test to compare RNFL overall, GCC average and ONH rim volume measurements pre-and post-dilation between groups

	Glaucoma patients			Healthy subjects		
	Pre-dilation	Post-dilation	*p*	Pre-dilation	Post-dilation	*p*
RNFL overall (μm)	80 ± 15	80 ± 16	0.87	99 ± 12	98 ± 14	0.70
GCC average (μm)	81.3 ± 13.4	81.1 ± 13.1	0.92	92.1 ± 6.7	91.5 ± 7	0.72
ONH rim volume (mm^3^)	0.05 ± 0.11	0.04 ± 0.07	0.74	0.11 ± 0.1	0.04 ± 0.07	0.36

RNFL = retinal nerve fibre layer; GCC = ganglion cell complex; ONH = optic nerve head

Mean quality scores (SSI RNFL, SSI GCC and SSI ONH) pre- and post-dilation are shown in Table 2. SSI tended to decrease after dilation but the difference wasn’t statistically significant.

**Table 2:** Student *t* test to compare SSI RNFL, SSI GCC and SSI ONH values pre- and post-dilation between groups

	Glaucoma patients			Healthy subjects		
	Pre-dilation	Post-dilation	*p*	Pre-dilation	Post-dilation	*p*
SSI RNFL	54.3 ± 10.3	51.7 ± 18.1	0.12	57 ± 10	54 ± 9.3	0.2
SSI GCC	58 ± 9.5	57 ± 8	0.55	60.9 ± 8.1	58.8 ± 7.3	0.3
ONH rim volume (mm^3^)	48.5 ± 7.6	46.6 ± 7.2	0.16	51.5 ± 8.9	50.4 ± 8.3	0.59

RNFL = retinal nerve fibre layer; GCC = ganglion cell complex; ONH = optic nerve head

There was not a statistically significant correlation between the measurements variability pre and post dilation and the quality score neither for the glaucoma group nor for the control group, as shown in Table 3.

**Table 3:** Pearson’s correlation coefficient (r) between the variability of pre- and post-dilation of all measurements and the mean quality score of the two measurements

	Glaucoma patients		Healthy subjects	
	*r*	*p*	*r*	*p*
SSI RNFL pre dilation-post dilation/mean RNFL overall	0.18	0.16	0.20	0.14
SSI GCC pre dilation-post dilation/mean GCC average	−0.19	0.14	0.02	0.86
SSI ONH pre dilation-post dilation/mean ONH rim volume	0.003	0.97	−0.06	0.68

RNFL = retinal nerve fibre layer; GCC = ganglion cell complex; ONH = optic nerve head

## Discussion

In this study the influence of pupil dilation on SD-OCT measurements and quality score has been investigated in glaucoma patients and healthy subjects.

The parameters considered in the analysis were RNFL overall, GCC average and ONH rim volume.

None of these measurements differed before and after pupil dilation either in glaucoma patients or healthy individuals.

The quality score tended to get slightly worst in both groups but the worsening was not statistically significant.

As expected, the measurements variability of all parameters pre- and post-dilation was not related to the quality score as shown by the Pearson’s coefficient.

While studies about the influence of pupil dilation on TD-OCT measurements gave opposing results [12–15], the few reports focused on the influence of pupil dilation on measurements performed by SD-OCTs and especially by the SD-OCT RTVue-100 give similar results concluding that measurements are not affected by pupil size [16].

For example, Garas et al. [17] investigated the influence of several factors, including pupil dilation, on the reproducibility of RNFL thickness and ONH measurements as performed with the SD-OCT RTVue-100 in healthy, ocular hypertensive and glaucomatous patients. Even if the authors discarded all poor quality images and included in the analysis only images with SSI > 45, their results agree with those of our study.

Massa et al. [18] compared the RNFL measurements in glaucoma patients and healthy subjects before and after dilation using a different SD-OCT, Cirrus HD-OCT Model 4000 (Carl Zeiss Meditec Inc.). Average thickness, quadrant thickness, and clock hour thickness measurements were compared as well as the quality score and no differences were found. Same results were obtained by Savini et al. [19].

We decided to compare only RNFL overall, GCC average and ONH rim volume because these parameters are commonly used in clinical practice.

Mwanza et al. [20] showed that also macular choroidal thickness measured by enhanced depth imaging optical coherence tomography acquisition mode on a Spectralis OCT device (Heidelberg Engineering, Heidelberg, Germany) didn’t show statistically difference pre- and post- dilation in healthy individuals and glaucoma patients.

Therefore, these studies confirm that the spectral-domain technology provides faster acquisition, better resolution, and improved visualization of retinal morphology as well as high quality scan, regardless the pupil size.

The main advantage of this technology is due to the use of an on-axis line camera to scan across the fundus and to construct the image from the respective lines taken, while the time domain uses an offaxis CCD camera, and, therefore, needs a wider pupil to allow the rays from the retina to pass.

The finding that both the quality of the scans and the measurements are similar before and after pupil dilation may represent an important advantage in clinical practice considering also that pupil dilation is time consuming, induces visual complaints, and may not be achievable in some patients.

Our study has important limitations: we included relatively young patients, without media opacities, very good BCVA and not taking miotics.

## Conclusions

In conclusion, our findings confirm that RNFL, GCC and ONH measurements as performed by the SD-OCT RTVue-100 are similar with undilated pupil and after pharmacological mydriasis, and that high quality images can be obtained without pupil dilation.

## Abbreviations

BCVA: best corrected visual acuity
GCC: ganglion cell complex
MD: mean deviation
OCT: optical coherence tomography
ONH: optic nerve head
PSD: pattern standard deviation
RNFL: retinal nerve fibre layer
SD-OCT: spectral-domain optical coherence tomography
SSI: signal strength index
TD-OCT: time-domain optical coherence tomography
VF: visual field
